# OP80 Cost-Utility Analysis Of Glucocorticoids Versus Standard Care For The Treatment Of Knee Osteoarthritis – Health Payer Perspective

**DOI:** 10.1017/S0266462325101347

**Published:** 2025-12-29

**Authors:** Danielle Stringer, Magdalena Moshi, Alvin Atlas, John Rathbone, Thomas Vreugdenburg

## Abstract

**Introduction:**

Knee osteoarthritis (OA) is a prevalent and debilitating condition with a significant impact on patient quality of life and healthcare costs. Intra-articular glucocorticoid injections (IAGI) are used in Switzerland for conservative management. A systematic literature review identified one existing economic evaluation on this topic globally. Therefore, the aim of this study was to evaluate the cost effectiveness of IAGI in knee OA (relative to standard care) within the Swiss healthcare context.

**Methods:**

A cost-utility analysis was conducted using a healthcare payer perspective. Health outcomes were measured using quality-adjusted life year (QALY) estimates. Clinical outcomes were mapped into a preference-based utility measure. The clinical data showed IAGI only improved pain at one month but not beyond. Only direct costs in 2024 CHF were considered. A time horizon of six months was used to capture differences in costs and outcomes. Given the short-term clinical improvement, only a single IAGI injection was modeled. Both costs and outcomes were discounted at three percent per annum. Both deterministic sensitivity analyses (DSA) and probabilistic sensitivity analyses (PSA) were conducted. The research project was funded by the Swiss Federal Office of Public Health.

**Results:**

The base case incremental cost-effectiveness ratio for IAGI in knee OA was CHF12,456 (USD15,735) per QALY gained. PSA showed a 71.9 percent probability of cost effectiveness at a hypothetical willingness-to-pay (WTP) threshold of CHF50,000 (USD63,164) per QALY gained and 75.0 percent at CHF100,000 (USD126,322). There was a 22.0 percent chance that IAGI is dominated by standard care (i.e., IAGI is more costly and less effective). The DSA identified the key model drivers as IAGI administration costs and changes in health-related quality of life post-treatment.

**Conclusions:**

IAGI for knee OA appeared to be a cost-effective adjunct to standard care under hypothetical thresholds of CHF50,000 (USD63,164) and CHF100,000 (USD126,322) per QALY gained. Incorporating both DSA and PSA allowed for a robust exploration of uncertainties, highlighting critical drivers of cost effectiveness. Future research should refine long-term effectiveness estimates, particularly the impact of IAGI on progression to joint replacement, and evaluate real-world outcomes to ensure optimal resource allocation.

